# Gender Differences in Statin Discontinuation and Adherence Among Privately Insured People with HIV in the USA

**DOI:** 10.1007/s11606-025-10164-x

**Published:** 2026-01-23

**Authors:** Thibaut Davy-Mendez, Alan C. Kinlaw, Shelby Tungate Lopez, N. Lance Okeke, Michelle Floris-Moore, Joseph J. Eron, Christy L. Avery, Stephen A. Berry, Heidi M. Crane, Carol E. Golin, Sonia Napravnik, Ross J. Simpson

**Affiliations:** 1 Division of Infectious Diseases, School of Medicine, University of North Carolina at Chapel Hill, Chapel Hill, NC USA; 2 Department of Epidemiology, Gillings School of Global Public Health, University of North Carolina at Chapel Hill, Chapel Hill, NC USA; 3 Division of Pharmaceutical Outcomes and Policy, Eshelman School of Pharmacy, University of North Carolina at Chapel Hill, Chapel Hill, NC USA; 4 Cecil G. Sheps Center for Health Services Research, University of North Carolina at Chapel Hill, Chapel Hill, NC USA; 5 UNC Medical Center, UNC Health, Chapel Hill, NC USA; 6 Division of Infectious Diseases, School of Medicine, Duke University, Durham, NC USA; 7 Department of Population Health Sciences, School of Medicine, Duke University, Durham, NC USA; 8 Division of Infectious Diseases, School of Medicine, Johns Hopkins University, Baltimore, MD USA; 9 Division of Allergy and Infectious Diseases, University of Washington, Seattle, WA USA; 10 Department of Health Services, University of Washington, Seattle, WA USA; 11 Division of General Medicine and Clinical Epidemiology, School of Medicine, University of North Carolina at Chapel Hill, Chapel Hill, NC USA; 12 Department of Health Behavior, Gillings School of Global Public Health, University of North Carolina at Chapel Hill, Chapel Hill, NC USA; 13 Division of Cardiology, School of Medicine, University of North Carolina at Chapel Hill, Chapel Hill, NC USA

**Keywords:** HIV, Statin, Adherence, Cardiovascular disease

## Abstract

**Background:**

People with HIV (PWH), particularly women, have a high cardiovascular disease (CVD) burden compared to the general population. There is little evidence describing statin adherence among PWH, which could inform interventions to reduce CVD disparities.

**Objective:**

Observational cohort of privately insured PWH under age 65 who initiated statin therapy during 2015–2022 in MarketScan data.

**Main Measures:**

We used outpatient pharmacy claims to examine (1) statin discontinuation, defined as a gap > 90 days, and (2) proportion of days covered (PDC) by a statin in 90-day intervals. We estimated hazard ratios (HRs) using Cox models to compare discontinuation rates and prevalence ratios (PRs) from log-binomial regression to compare the probability of having low adherence (PDC < 80%) between women and men. We adjusted for potential confounding by demographic and clinical factors and accounted for repeated PDC measures.

**Key Results:**

We included 9522 PWH who initiated a statin (median age 52 years, 17.3% women). Overall, 50.0% of PWH had statin discontinuation within 2 years, and 26.5% had low adherence during statin therapy. Within 2 years, 59.0% of women and 48.1% of men had experienced discontinuation (adjusted HR 1.30 (95% CI, 1.20–1.41)). Among PWH remaining on statins, low adherence (PDC < 80%) was more common among women (34.1%) than men (25.2%) (adjusted PR 1.29 (1.22–1.35)).

**Conclusions:**

PWH had high statin discontinuation rates, and a quarter had low adherence. Compared to men, women were both more likely to discontinue and have lower adherence. Efforts are needed to address statin adherence barriers to prevent CVD in PWH, especially in women with HIV.

**Supplementary Information:**

The online version contains supplementary material available at 10.1007/s11606-025-10164-x.

## INTRODUCTION

Cardiovascular disease (CVD) prevention in people with HIV (PWH) is a clinical and public health priority in the USA. Despite improved survival due to antiretroviral therapy,^1–3^ PWH have a persistently elevated CVD risk compared to people without HIV, with 9 fewer years of CVD-free life expectancy.^4–6^ Myocardial infarction (MI) and stroke rates are 21–76% higher for PWH than for people without HIV.^4,7–13^ HIV-related disparities are even more pronounced among women, with CVD rates that are up to twice as high for women with HIV vs. without HIV.^8,14,15^ The prevalence of MI is predicted to increase from 3.3% in 2020 to 8.1% in 2030 for PWH.^16^ Among PWH who do not inject drugs, heterosexual White, Black, and Hispanic women are predicted to have a higher MI prevalence in 2030 than almost every other demographic subgroup.^16^

Excess CVD risk in PWH is driven in part by HIV-related inflammation and a high prevalence of traditional CVD risk factors such as smoking, hypertension, and hyperlipidemia.^17,18^ Consequently, 40–60% of PWH are eligible for statin therapy per American College of Cardiology/American Heart Association (ACC/AHA) guidelines.^19–21^ In addition, the REPRIEVE trial recently demonstrated a 35% CVD reduction with pitavastatin in PWH at low-to-moderate risk, leading US guidelines to recommend moderate-intensity statin therapy for all PWH aged 40–75 with 10-year CVD risk between 5 and < 20%.^22,23^ However, only half of PWH on statins have cholesterol levels at target,^24–29^ which is lower than in people without HIV.^24,30–36^

In the general population, statin non-adherence is an important driver of poor cholesterol control, as over 60% of patients on a statin take it < 80% of the time, and over 30% of patients discontinue their statin within a year of initiation, with differences noted between men and women.^19,37–40^ Few studies have examined statin adherence among PWH, primarily focusing on differences between PWH and people without HIV, with data largely pre-dating the 2013 ACC/AHA guidelines.^24,41–43^ Little is known about statin adherence among PWH in more recent years, or about gender differences in statin adherence that could contribute to CVD gender disparities in PWH. We leveraged a nationwide claims database to investigate gender differences in statin discontinuation and adherence level among privately insured PWH.

## METHODS

### Data Source and Study Population

Data came from MarketScan Commercial Claims and Encounters databases (©2025 Merative; All rights reserved), which contain claims from participating employer-based commercial health insurance payers in the USA. Data include diagnoses, procedures, and outpatient pharmacy dispensations. We identified PWH as meeting both of the following criteria within 365 days of each other, in any order: (1) ≥ 1 dispensed antiretroviral, excluding agents used for HIV pre-exposure prophylaxis and hepatitis B virus treatment, and (2) either ≥ 1 outpatient HIV diagnosis, excluding claims associated with an HIV screening procedure code, or ≥ 1 HIV hospital discharge diagnosis in any position (see Supplementary Tables 1–3 for codes used). PWH who met the antiretroviral criteria but not the diagnosis criteria (e.g., those on post-exposure prophylaxis who did not become HIV positive) were not included. PWH became eligible on the date both criteria were met. We selected this definition after reviewing published algorithms used to identify PWH in MarketScan data (Supplementary Table 4).

We identified PWH initiating a statin between 2015 and 2022, defined as a new outpatient statin dispensation based on National Drug Codes (Supplementary Table 5), with no evidence of statin use in the past 6 months, while being continuously enrolled in a health plan with pharmacy benefits. For PWH with > 1 statin initiation episode during the study period, we included only the first episode. To focus on PWH prescribed statins for primary prevention, we excluded those with a history of CVD, defined using diagnosis and procedure codes (Supplementary Table 7).

This study was determined to be exempt from review by the Institutional Review Board of the University of North Carolina at Chapel Hill.

### Study Measures

We examined two outcomes: statin discontinuation and adherence level. We determined periods of statin use based on dispensation dates and the number of days supplied. If there was an overlap in dispensations of the same medication, the start date of the new dispensation was shifted to the end date of the prior one. However, if a patient had a dispensation for a new statin agent, it was assumed that the patient was instructed to initiate the new one immediately.

Statin discontinuation was defined as the first gap > 90 days in statin use. Discontinuation date was 90 days after the last date of statin use before the gap > 90 days. For discontinuation analyses, person-time was censored at the first gap in health plan coverage or December 31, 2022, whichever occurred earlier.

Adherence was measured using the proportion of days covered (PDC) calculated in 90-day intervals and categorized as low (< 80%) vs. high (≥ 80%), based on prior literature on chronic medication adherence including lipid-lowering therapy.^44^ We also conducted sensitivity analyses using < 70% and < 90% as thresholds of low adherence. In PDC analyses, person-time was censored at the earliest of the first gap in health plan coverage, December 31, 2022, or statin discontinuation, defined as above. We censored person-time after statin discontinuation to avoid our results being overly influenced by PWH who no longer had medication on hand and whose adherence could only be 0%.

We selected covariates that might differ between men and women and be strong drivers of statin adherence, including via patient motivation or the experience of side effects. These covariates were age, US census region of residence, calendar year of statin initiation, statin intensity (per agent and dose ^19^), use of a protease inhibitor (PI) or cobicistat-boosted antiretroviral, comorbidities (hypertension, hypercholesterolemia, diabetes mellitus, chronic kidney disease stage ≥ 3), and mental health and substance use disorders. Race/ethnicity data are not available in MarketScan. We selected comorbidities that have been shown to vary in prevalence by gender among PWH and that could potentially affect statin adherence, including via CVD risk perception.^45,46^ Comorbidities were defined as having either ≥ 1 inpatient or ≥ 2 outpatient diagnoses within 365 days of each other (Supplementary Table 6). This approach is used by the Centers for Medicare and Medicaid Services Chronic Conditions Data Warehouse and increases specificity by not counting patients with a single outpatient diagnosis.^47^ We did not use medication data to define comorbidities to avoid undercapturing PWH not taking treatment for their conditions.

In discontinuation analyses, all covariates were measured at statin initiation using all claims up to the initiation date. In PDC analyses, age, time since statin initiation, PI or cobicistat use, and history of comorbidities were time-updated and measured at the start of every 90-day interval.

### Statistical Analysis

Overall and stratified by gender, we estimated the cumulative incidence of discontinuation at 1 year and 2 years from Kaplan-Meier curves, accounting for PWH who were censored during follow-up. We used Cox proportional hazards models to estimate hazard ratios (HRs) comparing discontinuation rates by gender, adjusted for all baseline covariates.

We plotted 90-day PDC over time by gender and estimated differences in the probability of having low adherence (PDC < 80%) using prevalence ratios (PRs) estimated with log-binomial regression models, using generalized estimating equations (GEE) to account for PWH contributing more than one 90-day interval to the analysis, adjusted for all covariates and time since statin initiation. In a sensitivity analysis, we censored all PWH at 3 years after statin initiation to evaluate the potential impact on our findings of a small number of patients having very long follow-up. All analyses were conducted in SAS 9.4 (SAS Institute, Cary, NC).

## RESULTS

### Study Sample

The 9522 included PWH were 83% men and a median of 52 years old (IQR 46–57) (Table 1). Compared to men, women were more likely to reside in the Northeast (21% vs. 14%) or the South (69% vs. 60%) and to have hypertension (56% vs. 42%) or diabetes mellitus (28% vs. 18%).

**Table 1:** Characteristics of 9522 People with HIV (PWH) Initiating a Statin, Overall and Stratified by Gender, MarketScan Commercial Claims and Encounters Databases, 2015–2022

Characteristic at statin initiation	All PWH *N* = 9522	Men *N* = 7870	Women *N* = 1652
Age, years	52 (46, 57)	52 (46, 57)	53 (47, 58)
Region of residence			
Northeast	1478 (16%)	1131 (14%)	347 (21%)
North Central	909 (10%)	815 (10%)	94 (6%)
South	5811 (61%)	4682 (60%)	1129 (69%)
West	1305 (14%)	1228 (16%)	77 (5%)
Calendar year			
2015–2017	3065 (32%)	2565 (33%)	500 (30%)
2018–2019	2721 (29%)	2248 (29%)	473 (29%)
2020–2021	3736 (39%)	3057 (39%)	679 (41%)
Medical comorbidities			
Hypertension	4218 (44%)	3296 (42%)	922 (56%)
Hypercholesterolemia	1053 (11%)	869 (11%)	184 (11%)
Diabetes mellitus	1895 (20%)	1432 (18%)	463 (28%)
Chronic kidney disease	396 (4%)	325 (4%)	71 (4%)
Psychiatric history			
Mental health disorder	2362 (25%)	1958 (25%)	404 (24%)
Alcohol use disorder	277 (3%)	246 (3%)	31 (2%)
Other substance use disorder	1217 (13%)	998 (13%)	219 (13%)
Any PI or cobicistat use	3106 (33%)	2594 (33%)	512 (31%)
First statin agent			
Atorvastatin	5103 (54%)	4257 (54%)	846 (51%)
Rosuvastatin	2775 (29%)	2321 (29%)	454 (27%)
Pravastatin	1241 (13%)	988 (13%)	253 (15%)
Simvastatin	297 (3%)	217 (3%)	80 (5%)
Other*	106 (1%)	87 (1%)	19 (1%)
Statin intensity			
Low	1692 (18%)	1340 (17%)	352 (21%)
Medium	6110 (64%)	5098 (65%)	1012 (61%)
High	1720 (18%)	1432 (18%)	288 (17%)

Numbers are *N* (%) or median (interquartile range)

^*^Includes 51 PWH initiating pitavastatin, 45 lovastatin, and 10 fluvastatin

### Statin Discontinuation

**Figure 1:**
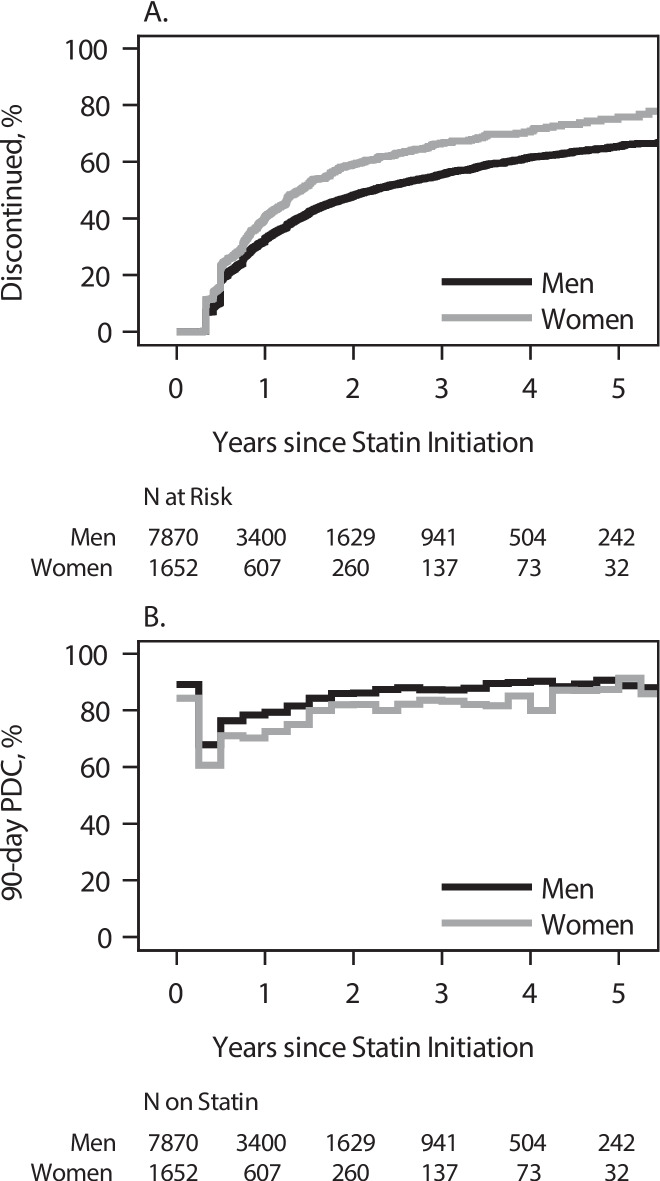
Time to discontinuation > 90 days (**A**) and 90-day proportion of days covered (PDC) by a statin (**B**), stratified by gender, among privately insured people with HIV initiating a statin in the MarketScan Databases, 2015–2022. Lines in **A** represent the cumulative incidence of discontinuation. Lines in **B** represent PDC over each 90-day interval. Numbers below each panel are the number of people at a given time point who have not discontinued statin therapy.

Among all PWH, 1-year cumulative incidence of statin discontinuation (> 90-day gap) was 33.9% (95% CI 32.8–35.0%) and 2-year cumulative incidence was 50.0% (48.7–51.3%). Women were more likely to experience discontinuation, with 59.0% (55.9–62.2%) at 2 years since initiation, compared to 48.1% (46.7–49.5%) for men (Table 2, Fig. 1A). The adjusted HR comparing discontinuation rates for women vs. men was 1.30 (95% CI 1.20–1.41) (Table 2). Younger age and earlier calendar years were also associated with higher discontinuation rates, while having hypercholesterolemia, having a mental health disorder, and residing in a region other than the South were associated with lower discontinuation rates (Supplementary Table 8).

**Table 2:** Comparison of Statin Use Outcomes Between 1652 Women and 7870 Men with HIV Initiating a Statin, MarketScan Commercial Claims and Encounters Databases, 2015–2022

Gender	Two-year risk of discontinuation *	Unadjusted HR (95% CI)^†^	Adjusted HR (95% CI)^†, ‡^
Women	59.0% (55.9%, 62.2%)	1.33 (1.23, 1.44)	1.30 (1.20, 1.41)
Men	48.1% (46.7%, 49.5%)	1 (ref.)	1 (ref.)
Gender	Probability of low adherence^§^	Unadjusted PR (95% CI)^||^	Adjusted PR (95% CI)^||, ¶^
Women	34.1% (32.5%, 35.9%)	1.36 (1.29, 1.44)	1.29 (1.22, 1.36)
Men	25.0% (24.4%, 25.7%)	1 (ref.)	1 (ref.)

Abbreviations: *CI*, confidence interval; *HR*, hazard ratio; *PR*, prevalence ratio; *ref.*, referent

^*^First gap in statin use > 90 days

^†^Estimated from a Cox proportional hazards model

^‡^Adjusted for age, calendar year, US census region, statin intensity, use of a protease inhibitor or cobicistat-boosted antiretroviral therapy regimen, and history of hypertension, hypercholesterolemia, diabetes mellitus, chronic kidney disease stage 3 or greater, mental health disorders, alcohol use disorder, and other substance use disorder, all measured at statin initiation

^§^Low adherence was defined as having a 90-day proportion of days covered (PDC) < 80%

^||^Estimated from a log-binomial regression model with generalized estimating equations accounting for patients contributing more than one 90-day interval to the analysis

^¶^Adjusted for calendar year, US census region, and statin intensity, measured at statin initiation, and for time-updated age, use of a protease inhibitor or cobicistat-boosted antiretroviral therapy regimen, and history of hypertension, hypercholesterolemia, diabetes mellitus, chronic kidney disease stage 3 or greater, mental health disorders, alcohol use disorder, and other substance use disorder, measured at the start of every 90-day interval

### Statin Adherence

PWH contributed 12,172 person-years on a statin (10,317 and 1855 among men and women, respectively) that were divided into 90-day intervals for PDC analyses. The median person-years contributed by each PWH was 0.8 (0.4–1.7) among men and 0.7 (0.3–1.4) among women. Most PWH (89.6%) had at least one 90-day interval of follow-up before censoring.

Over all 90-day intervals among both men and women who were on a statin at some point in the past 90 days, the average 90-day PDC was 80.6% (95% CI 80.1–81.0%). The median 90-day PDC was 100.0% (IQR 75.6–100.0%; range 0.0–100.0%). PWH had low adherence (PDC < 80%) in 26.5% (95% CI 25.8–27.1%) of time intervals. Among those with PDC < 80%, the median PDC was 33.3% (IQR 0.0–63.3%; range 0.0–79.7%).

Among PWH who were on a statin at some point in the past 90 days, 90-day PDC was lower for women compared to men for the full study period, at 75.2% (95% CI 73.9–76.4) and 81.6% (81.1–82.1%), respectively, and across most time intervals in the study period (Fig. 1B). For both men and women, PDC decreased from the first to the second 90-day interval and subsequently increased over time with longer statin use duration. Over all 90-day intervals, among PWH who were on a statin at some point in the past 90 days, women were more likely to have low statin adherence (90-day PDC < 80%) compared with men, with 34.1% (95% CI 32.5–35.9%) of women and 25.0% (24.4–25.7%) of men having low adherence. Compared to men, women were 1.29 (95% CI 1.22–1.36) as likely to have low adherence over all 90-day intervals in adjusted analyses (Table 2).

Younger age was associated with a higher probability of low adherence, while earlier calendar years, having hypercholesterolemia, having a mental health disorder, and residing in the North Central or West vs. South regions were associated with a lower probability of low adherence (Supplementary Table 8).

### Sensitivity Analyses

Gender differences in analyses using 90% or 70% as a threshold of low adherence were similar to the primary findings using 80% (Supplementary Table 9). In addition, in a sensitivity analysis censoring all PWH at 3 years to account for the drop in sample sizes over time, we found estimates similar to the primary findings (Supplementary Table 10).

## DISCUSSION

Among > 9000 privately insured PWH initiating statins, we found that one-third discontinued within a year of initiation. Over a quarter of PWH on statins had adherence levels < 80%, most of whom had very low adherence levels. After adjusting for demographic and clinical factors, we found that women were more likely than men to have suboptimal statin use, with a 30% higher discontinuation rate and a 29% higher probability of having adherence < 80%.

Only a few studies have examined statin discontinuation and adherence among PWH. Among PWH initiating statins in the Kaiser Permanente Northern California health system 1996–2005, the average adherence level in the first year was 84%, with close to half having adherence < 96%.^24^ Among Medicaid beneficiaries with HIV initiating statins 2001–2010, only about half had very high statin adherence levels, defined as > 90% adherence, averaged over the first 2 years of statin use.^42^ A study of MarketScan commercial claims data from 2007 to 2016 reported a 1-year statin discontinuation rate of 27% among PWH, using the same 90-day definition as our analyses.^41^ The slightly higher discontinuation rate in our study (34%) might reflect the expansion of statin use to more patients at lower CVD risk following the 2013 ACC/AHA guideline changes.^21^ This MarketScan study reported average adherence levels of 68% over the first year of statin use, consistent with the low adherence that we observed in the first year.

Our study extends prior work in several ways. With data from 2015 to 2022, we provided the most recent report on statin use among PWH in the USA, capturing the period after the 2013 ACC/AHA guidelines, which substantially expanded statin eligibility in PWH, and a shift in the use of antiretroviral drugs that might interact with statins, which has important implications for statin prescribing practices and patient adherence.^21,43,48^ By estimating statin adherence levels in 90-day intervals, rather than averaged over longer periods, we were able to identify patterns over the course of statin therapy. Adherence levels were low in the first year of statin therapy, and, as the proportion of PWH who had discontinued increased over time, there was a steady increase in adherence levels among PWH who remained on statins.

Our findings indicate that statin persistence and adherence are worse for women vs. men with HIV. Among PWH, antiretroviral adherence has generally been found to be higher for women compared to men, though some studies have found the opposite or no association.^49–53^ In the general population, statin adherence has been reported to be higher for men than women, and for white vs. non-white patients.^39,40^ Mechanisms for these differences are not fully understood but could be partly explained by factors such as financial barriers (e.g., lower income, poor or no insurance coverage), knowledge and beliefs about statins and CVD, and social influences (e.g., patient-provider relationship, experiences and advice from others).^54,55^ Statin adherence is also higher for patients with more CVD risk factors,^39^ which may reflect higher levels of risk perception motivating adherence. Among PWH, qualitative evidence has shown that patients may not adhere to statins and other CVD preventatives due to low CVD risk perception, lack of knowledge about CVD and medications to prevent it, concerns about side effects and drug-drug interactions, or pill fatigue from long-term antiretroviral therapy.^56–58^

We did not have data on the reason for statin discontinuation, and it is possible that it was clinically indicated for some PWH in our study. Clinical trials have demonstrated that statins are safe, with very low rates of myopathy and rhabdomyolysis, and little effect on less severe pain.^59–61^ Nonetheless, studies in the general population have reported that muscle pain was the main reason reported for statin discontinuation, and women were more likely to discontinue because of side effects. ^62,63^

Our findings highlight the need for interventions to promote long-term statin use and adherence in PWH, particularly women. The high discontinuation rates and low adherence levels in the first year of statin therapy suggest that intervening at statin initiation or soon after might be an effective strategy. In the general population, various interventions, including patient reminders, counseling, and education, have been proven effective to improve statin adherence and cholesterol control ^64,65^. Evidence-based interventions tailored to PWH are lacking. A recent trial in PWH demonstrated a significant decrease in non-HDL cholesterol with nurse-led care coordination, treatment protocols, and adherence counseling.^66^ Other interventions, particularly focused on clinic- and provider-level factors and implementation strategies, are being evaluated to increase statin use in PWH.^67,68^ But more interventions addressing patient-level barriers to statin adherence in PWH are needed. Furthermore, the new guidelines expanding statin eligibility for PWH over 40 will likely lead to an increased uptake among patients with lower CVD risk, who may have different statin adherence levels ^21,23^. Continuing to evaluate adherence among PWH will be essential to ensure all eligible patients experience the clinical benefits of statin therapy.

Given PWH’s high CVD burden, promoting continued statin use at high levels of adherence is particularly important in this population. Based on the REPRIEVE trial in PWH at low-to-intermediate CVD risk, the number needed to treat (NNT) with a statin to prevent one major atherosclerotic cardiovascular event over 5 years is 34 for PWH over 40 with a 10-year CVD risk of 10–19%, and 53 for those with a CVD risk of 5–10%.^22,23^ Studies suggest NNTs are substantially higher for similar people without HIV,^69–72^ likely in part because CVD risk scores underestimate CVD risk in PWH.^73,74^ PWH are therefore an important population to target for CVD prevention efforts.

### Strengths and Limitations

Our study included a large sample size and 8 years of recent, nationwide claims data. We were able to adjust for important confounders including age, statin intensity, use of antiretrovirals with potential for drug-drug interactions with statins, and medical and psychiatric comorbidities. However, our findings among privately insured PWH may not be generalizable to all PWH in the US, though about 35% of PWH in the USA have private insurance.^75^ Because MarketScan does not capture sex and gender identity separately and only includes values “male” and “female,” we were not able to identify transgender PWH in our study, who may have different barriers to adherence. We did not have data on automatic refills or home delivery for prescriptions, which could affect adherence. We also lacked data on social determinants of health, which could also affect adherence, even among privately insured PWH. Our analyses measured adherence levels in 90-day intervals of follow-up and did not examine patients with low adherence over multiple intervals, which future studies should investigate. Finally, our study only included PWH who had ≥ 1 dispensation for statins and does not capture PWH with a statin prescription that was never filled, i.e., primary non-adherence. Evidence from the general population has shown that up to 28% of initial statin prescriptions are never filled,^76,77^ and research is needed on primary non-adherence in PWH.

In a large sample of privately insured people with HIV in the USA initiating statin therapy, over a third experienced a statin discontinuation within 1 year, and over a quarter of those remaining on statins had low adherence. Women with HIV were more likely to discontinue and have low adherence compared to men with HIV. Continued efforts are needed to promote long-term statin use and adherence in PWH, particularly among women with HIV, to reduce the high CVD burden in this population.

## Supplementary Information

Below is the link to the electronic supplementary material.

ESM 1 (DOCX 78.4 KB)

## Data Availability

Data Availability Statement: MarketScan databases can be accessed via Merative.
