# Supplementary material for: Gender Differences in Statin Discontinuation and Adherence Among Privately Insured People with HIV in the USA
Source: J Gen Intern Med. 2026 Jan 23;41(10):2873–81. doi: 10.1007/s11606-025-10164-x (PMC13189131; doi:10.1007/s11606-025-10164-x)
Supplement: Supplementary file 1 — (DOCX 78.4 KB) [file 11606_2025_10164_MOESM1_ESM.docx]

**SUPPLEMENTARY APPENDIX**

**Title: Gender differences in statin discontinuation and adherence among privately insured people with HIV in the United States**

**Authors: Thibaut DAVY-MENDEZ, PhD Alan C. KINLAW, PhD Shelby TUNGATE LOPEZ, PharmD, CPP, BCACP, MPA, N. Lance OKEKE, MD, MPH, Michelle FLORIS-MOORE, MD, MS, Joseph J. ERON, MD, Christy L. AVERY, MPH, PhD, Stephen A. BERRY, MD, PhD, Heidi M. CRANE, MD, MPH, Carol E. GOLIN, MD, Sonia NAPRAVNIK, PhD, Ross J. SIMPSON, Jr., MD, PhD**

**Table of Contents**

[Supplemental Table 1. National Drug Codes (NDCs) for antiretroviral medications included and excluded when identifying people with HIV. 1](#_Toc190347571)

[Supplemental Table 2. International Classification of Diseases (ICD) codes for HIV. 5](#_Toc190347572)

[Supplemental Table 3. Procedure codes used to exclude claims associated with HIV screening. 6](#_Toc190347573)

[Supplemental Table 4. Definitions used in prior studies to identify people with HIV in claims data. 7](#_Toc190347574)

[Supplemental Table 5. National Drug Codes (NDCs) used to identify statins. 9](#_Toc190347575)

[Supplemental Table 6. International Classification of Diseases (ICD) codes used to identify patients with medical and psychiatric health conditions. 12](#_Toc190347576)

[Supplemental Table 7. International Classification of Diseases (ICD) codes and procedure codes used to identify patients with a history of cardiovascular disease. 14](#_Toc190347577)

[Supplemental Table 8. Sensitivity analyses using different thresholds of proportion of days covered (PDC) to define low adherence. 16](#_Toc190347578)

[Supplemental table 9. Sensitivity analysis censoring all people with HIV after three years since statin initiation. 17](#_Toc190347579)

[References 18](#_Toc190347580)

# Supplementary Table 1. National Drug Codes (NDCs) for antiretroviral medications included and excluded when identifying people with HIV.

| Medication | 9-Digit NDC | Exclude |
| --- | --- | --- |
| 3TC | 001730470, 001730471, 001730662, 001730663, 001730714, 003785168, 003785169, 003785170, 004034977, 009046583, 101350605, 216950367, 317220001, 317220752, 317220753, 317220754, 333420001, 333420002, 353560065, 353560066, 497020203, 497020204, 497020205, 499990119, 502680459, 502680460, 507420623, 507420624, 512930832, 529590508, 545694221, 545694333, 545695479, 545695501, 548380566, 548683693, 548685416, 550452308, 558870750, 572370274, 580160689, 580160795, 603461016, 604290353, 604290354, 605053250, 605053251, 605053252, 606870362, 626821016, 643800710, 643800711, 658620025, 658620026, 658620055, 658620552, 658620553, 669930478, 672630258, 672630384, 680306060, 680306064, 680840578, 681800602, 681800603, 682589107, 682589108, 690970166, 690970167 | Yes |
| 3TC, ABC | 000935382, 001730742, 353560109, 355730402, 355730430, 422910115, 423850962, 497020206, 504360206, 545695594, 548685600, 658620335, 658620900, 669930482, 681800288, 682581988, 690970362, 707101049, 707711053 | No |
| 3TC, ABC, DTG | 497020231, 497020258, 500901606, 545696569 | No |
| 3TC, ABC, ZDV | 001730691, 353560116, 497020217, 545695191, 681800286, 682589158 | No |
| 3TC, DOR, TDF | 000065007 | No |
| 3TC, DTG | 497020246 | No |
| 3TC, EFV, TDF | 423850928, 423850929, 495020425, 495020475 | No |
| 3TC, TDF | 322280004, 495020450, 726060002 | No |
| 3TC, ZDV | 000935385, 001730595, 003785180, 165900061, 188370031, 216950846, 216950996, 234907087, 317220506, 317220739, 333420003, 422540381, 422910363, 430630346, 430630900, 497020202, 499990062, 500901419, 502680456, 504360597, 529590546, 532170052, 545694524, 545696484, 548684114, 550452856, 551755207, 552890389, 557000096, 557000304, 557000411, 558870231, 580160698, 607600385, 607600595, 619190061, 626821048, 636294872, 638740100, 643800707, 658620036, 658620597, 662670509, 663360563, 680307283, 680710908, 680714450, 680840416, 681150090, 681800284, 682581977, 682581993, 687887298, 713350141, 713350639, 713351916, 728650144, 762820115, 765191005 | No |
| ABC | 001210897, 001730661, 001730664, 003784105, 009046523, 009046874, 317220557, 317220562, 353560075, 497020221, 497020222, 502680049, 510790204, 545694883, 545695390, 545696351, 548684522, 605053583, 643800717, 649800405, 658620073, 658620089, 680840021, 690970514, 705181274, 728650167 | No |
| APV | 001730672, 001730679, 001730687, 545694813 | No |
| ATV | 000033622, 000033623, 000033624, 000033631, 000033638, 000935526, 000935527, 000935528, 009046875, 167140860, 167140861, 167140862, 317220653, 317220654, 317220655, 353560068, 353560114, 353560207, 423850920, 423850921, 423850922, 500901581, 514070171, 514070172, 514070173, 545695530, 545695532, 545695864, 548684854, 548684857, 548685838, 548984854, 597620408, 597620409, 597620410, 606870399, 658620710, 658620711, 658620712, 658620713, 672630230, 672961236, 682581984, 682581985, 682589142, 690970443, 690970444, 690970445, 690970446, 692381135, 692381136, 692381137, 692381138, 705181365, 705182832, 707101050, 707101051, 707101052, 707711591, 707711592, 707711593 | No |
| ATV, COBI | 000033641, 500901724, 545696595 | No |
| BIC, FTC, TAF | 619582501, 619582505 | No |
| CAB | 497020248, 497020264 | Yes |
| CAB, RPV | 497020240, 497020253 | Yes |
| COBI | 619581401 | No |
| COBI, DRV | 545696594, 596760575 | No |
| COBI, DRV, FTC, TAF | 596760800 | No |
| COBI, EVG, FTC, TAF | 500902279, 545696633, 619581901 | No |
| COBI, EVG, FTC, TDF | 545696352, 548686371, 619581201 | No |
| d4T | 000031964, 000031965, 000031966, 000031967, 000031968, 003785040, 003785041, 003785042, 003785043, 317220515, 317220516, 317220517, 317220518, 353560074, 353560285, 427990113, 545694053, 545694054, 545695387, 545695412, 545695480, 545696123, 545696206, 548683352, 548683353, 548683360, 548683448, 597621190, 597621191, 597621192, 597621193, 643760133, 649800354, 649800355, 649800356, 649800357, 658620046, 658620047, 658620111, 658620112, 672530761, 680840460, 682589126 | No |
| ddC | 000040220, 000040221, 545693877, 545694485, 548682499, 548682500, 550452207 | No |
| ddI | 000876614, 000876615, 000876616, 000876632, 000876633, 000876650, 000876651, 000876652, 000876653, 000876665, 000876671, 000876672, 000876673, 000876674, 003788886, 003788887, 003788888, 003788889, 005550588, 005550589, 005550590, 353560186, 353560259, 511292999, 545693657, 545694313, 545694514, 545694905, 545695176, 545695504, 545695642, 545695643, 548682502, 548684666, 548685464, 548685595, 625840046, 625840048, 658620310, 658620311, 658620312, 658620313, 680840431, 680840432 | No |
| DLV | 000093761, 000097576, 497020209, 497020210, 497020225, 545694562, 545695122, 545695602, 548684520, 630100020, 630100021, 672630458 | No |
| DOR | 000063069 | No |
| DRV | 353560113, 353560284, 500901327, 545695814, 545696086, 545696159, 545696366, 545696403, 548685631, 548685969, 548686369, 596760560, 596760561, 596760562, 596760563, 596760564, 596760565, 596760566, 672630590, 682581980, 682581986, 705181483 | No |
| DTG | 497020226, 497020227, 497020228, 497020255, 538081130, 545696419 | No |
| DTG, RPV | 497020242 | No |
| EFV | 000560470, 000560473, 000560474, 000560510, 003782233, 317220504, 353560069, 353560115, 425430889, 514070382, 545694611, 545695374, 548684668, 643800889, 649800406, 649800407, 658620049, 658620104, 658620105, 658620106, 658620774, 672630568, 682581979, 682589020, 682589021, 690970301, 705181100, 705182962, 705183258, 728650172 | No |
| EFV, FTC, TDF | 000935234, 155840101, 317220736, 333420138, 353560064, 423850915, 500900980, 504360101, 545695805, 548685643, 658620497, 682581990, 690970210, 705182905, 762820678 | No |
| ETV | 545696102, 545696230, 548685864, 548686368, 596760570, 596760571, 596760572, 602191720, 602191721, 602191722, 682581982, 692381720, 692381721, 692381722, 693150285, 693150286, 800050112, 800050113 | No |
| FOS | 001730721, 001730727, 003783520, 353560067, 497020207, 497020208, 545695550, 548684954, 633040583, 682581994 | No |
| FPV | 672630387 | No |
| FTC | 353560205, 545695521, 548684853, 619580601, 619580602, 690970642, 705181847, 705182906 | Yes |
| FTC, RPV, TAF | 500902340, 545696645, 619582101 | No |
| FTC, RPV, TDF | 504361101, 545696270, 548686360, 619581101, 682581989 | No |
| FTC, TAF | 619582002, 619582005, 705180230 | Yes |
| FTC, TDF | 000937607, 000937704, 003781930, 009047172, 167140534, 317220560, 333420106, 353560070, 422910439, 423850953, 425430719, 500900870, 500905957, 504360701, 514070112, 529590969, 545695588, 548685141, 550453481, 602192095, 605054202, 619190669, 619580701, 619580703, 619580704, 619580705, 636297581, 643800719, 658620354, 663360032, 672630260, 672961237, 672961433, 680712112, 681800287, 682581983, 690970209, 690970741, 692381527, 692381547, 692381548, 692381549, 692382092, 692382093, 692382094, 692382095, 707101364, 707101365, 707101366, 707101367, 707711620, 707711621, 707711622, 707711709, 713351844, 721890156, 721890227, 721890312, 762820677 | Yes |
| FTR | 497020250 | No |
| IBA | 620640122 | No |
| IDV | 000060570, 000060571, 000060573, 000060574, 165900064, 216950366, 353560139, 529590507, 545698620, 548684113, 551755209, 558870230, 580160699 | No |
| LPV, RTV | 000740522, 000741575, 000742605, 000743956, 000743959, 000746799, 004907028, 005271947, 216950362, 317220556, 317220603, 353560111, 353560112, 423850933, 423850934, 529590134, 529590968, 545695142, 545695525, 545695752, 545696199, 548684524, 548685566, 550453482, 552890931, 552890947, 663360624, 672630232, 680710748, 680712348, 682581972, 705180091 | No |
| MVC | 000690807, 000690808, 317220579, 317220580, 353560208, 353560209, 497020215, 497020216, 497020223, 497020224, 497020233, 497020235, 497020237, 497020260, 504360224, 545696143, 545696214, 548685809, 672630402 | No |
| NFV | 353560117, 499990431, 529590289, 545694543, 545695573, 548683947, 548685061, 551755208, 552890477, 607600010, 630100010, 630100011, 630100027, 662670514, 680307284 | No |
| NVP | 000540450, 000540459, 000543905, 000544647, 000548647, 003784050, 003784890, 003786950, 005970046, 005970047, 005970123, 005970129, 007815893, 139250500, 317220505, 333420004, 333420238, 353560071, 353560072, 425710131, 477810100, 477810317, 504360123, 519910331, 545694561, 545696236, 548683844, 548686370, 552890392, 604290298, 605053788, 605054058, 614420470, 643800709, 651620209, 658620027, 658620057, 658620932, 658620933, 672630434, 682581978, 690970403 | No |
| RAL | 000060227, 000060473, 000060477, 000063080, 000063603, 353560110, 500901085, 545696034, 548680117, 619190706, 663360214, 672961235, 680712113, 682581981 | No |
| RPV | 596760278 | No |
| RTV | 000540407, 000741940, 000742340, 000743333, 000743399, 000746633, 000749492, 317220597, 353560138, 538081119, 545694335, 545694613, 545694792, 545695656, 545696170, 548683782, 606870364, 606870420, 651620061, 658620687, 682581987, 683820696, 690970655 | No |
| SQV | 000040244, 000040245, 000040246, 545694242, 545694563, 545695664, 548683699, 548684110 | No |
| T20 | 000040380, 000040381, 353560206, 545695781 | No |
| TAF | 619582301 | Yes |
| TDF | 000937104, 009046821, 167140820, 317220535, 333420096, 353560073, 422910800, 423850901, 502680758, 538081128, 545695334, 548684669, 605054666, 606870366, 619580401, 619580403, 619580404, 619580405, 619580406, 621350466, 643800714, 658620421, 658620818, 658620819, 658620820, 672630455, 682581971, 682589003, 690760105, 690970533, 705181002 | Yes |
| TPV | 005970002, 005970003, 545695719 | No |
| ZDV | 000540052, 000810107, 000810108, 000810113, 000935530, 001730107, 001730108, 001730113, 001730501, 003786106, 004034049, 004907026, 005271905, 216950369, 317220509, 332610613, 427990403, 427990404, 497020211, 497020212, 497020213, 497020214, 499990386, 509620450, 509620452, 523430044, 523430045, 529590387, 529590509, 531040101, 545691772, 545694334, 545694538, 545696171, 548681974, 548682504, 550453549, 551754494, 580160690, 580160864, 588640462, 597623650, 603461015, 626821015, 633040920, 643760128, 643760129, 658620024, 658620048, 658620107, 672530109, 672530961, 672630514, 680306059, 680306065, 680840461, 680840462, 705181656 | No |

Abbreviations: 3TC, lamivudine; ABC, abacavir; APV, amprenavir; ATV, atazanavir; BIC, bictegravir; CAB, cabotegravir, COBI, cobicistat; d4T, stavudine; ddC, zalcitabine; ddI, didanosine; DLV, delavirdine, DOR, doravirine; DRV, darunavir; DTG, dolutegravir; EFV, efavirenz; ETV, etravirine; EVG, elvitegravir; FOS, fosamprenavir; FTC, emtricitabine; FTR, fostemsavir; IBA, ibalizumab; IDV, indinavir; LPV, lopinavir; MVC, maraviroc; NFV, nelfinavir; NVP, nevirapine; RPV, rilpivirine; RAL, raltegravir; RTV, ritonavir; SQV, saquinavir; T20, enfuvirtide; TAF, tenofovir alafenamide; TDF, tenofovir disaproxil fumarate; TPV, tipranavir; ZDV, zidovudine.

# Supplementary Table 2. International Classification of Diseases (ICD) codes for HIV.

| Diagnosis | Coding System | Code |
| --- | --- | --- |
| Human immunodeficiency virus [HIV] disease | ICD-9-CM | 042 |
| Asymptomatic human immunodeficiency virus [HIV] infection status | ICD-9-CM | V08 |
| Human immunodeficiency virus [HIV] disease | ICD-10-CM | B20 |
| Asymptomatic human immunodeficiency virus [HIV] infection status | ICD-10-CM | Z21 |

Abbreviations: ICD-9-CM; International Classification of Diseases, version 9, clinical modification; ICD-10-CM, International Classification of Diseases, version 10, clinical modification.

# Supplementary Table 3. Procedure codes used to exclude claims associated with HIV screening.

| Procedure | Coding System | Code |
| --- | --- | --- |
| Obstetric panel including HIV | CPT | 80081 |
| HTLV or HIV antibody, confirmatory test (eg, Western Blot) | CPT | 86689 |
| HIV-1, HIV-2, HIV-1 and HIV-2, single result (antibody) | CPT | 86701–86703 |
| HIV-1 (antigen) | CPT | 87390 |
| HIV-2 (antigen) | CPT | 87391 |
| HIV-1 and/or HIV-2, screening, EIA | HCPCS | G0432 |
| HIV-1 and/or HIV-2, screening, ELISA | HCPCS | G0433 |
| HIV-1 and/or HIV-2, screening, rapid antibody test | HCPCS | G0435 |
| HIV antigen/antibody, screening, combination assay | HCPCS | G0475 |

Abbreviations: CPT, Current Procedural Terminology; HCPCS, Healthcare Common Procedure Coding System.

# Supplementary Table 4. Definitions used in prior studies to identify people with HIV in claims data.

| Study | Definition |
| --- | --- |
| Alonso et al., 2019^1^ | Any of the following ICD-9-CM codes in any claim: 042 (HIV disease), 079.53 (HIV, type 2), 795.71 (non‐specific serologic evidence of HIV), or V08 (asymptomatic HIV infection status) |
| Crockett et al., 2021^2^ | One of the following:   - At least one hospitalization with a discharge diagnosis code for HIV (ICD-9-CM 042.x-044.x or V08, or ICD-10-CM B20.xx-B22.xx, B24.xx or Z21.xx) in any discharge diagnosis position - At least two pharmacy fills for ART no more than 365 days apart, excluding lamivudine, tenofovir, and emtricitabine which can be prescribed for hepatitis or pre-exposure prophylaxis |
| Gallant et al., 2017^3^ | At least one inpatient or outpatient claim (defined as a claim that was not for a procedure that is used to confirm an HIV diagnosis) associated with an ICD-9-CM diagnosis code for HIV (codes 042.xx, 079.53, and v08.xx) |
| King et al., 2020^4^ | At least one inpatient or outpatient medical claim of HIV diagnosis, defined using diagnostic, procedural, and drug codes |
| Kong et al., 2019^5^ | At least one of the following:   - At least two claims with an ICD-9-CM diagnosis of HIV (042, 795.71, V08, 079.53) in any position (primary or secondary) - One claim with an HIV diagnosis with at least one claim for ART |
| Rosenson et al., 2020^6^ | At least one of the following:   - ≥1 hospitalization with a discharge diagnosis code for HIV in any position. Discharge diagnosis codes for HIV included an International Classification of Diseases, Ninth Revision (ICD‐9), code of 042.x to 044.x or V08 or an ICD‐10 code of B20.xx to B22.xx, B24.xx, or Z21.xx - ≥2 pharmacy claims for ART |
| Siddiqui et al., 2022^7^ | At least one diagnosis code for HIV (042 [ICD-9-CM] or B20 [ICD-10-CM]) |
| Siddiqui et al., 2023^8^ | Both of the following:   - At least one hospitalization with a discharge diagnosis code in for HIV (ICD-9-CM 042.x to 044.x or V08 or ICD-10 B20.xx to B22.xx, B24.xx, or Z21.xx) - At least 2 pharmacy fills for ART no more 365 days apart, excluding lamivudine, tenofovir disoproxil fumarate, and tenofovir alafenamide, and emtricitabine/tenofovir disoproxil fumarate and emtricitabine/tenofovir alafenamide combinations |

Abbreviations: ART, antiretroviral therapy. ICD-9-CM; International Classification of Diseases, version 9, clinical modification; ICD-10-CM, International Classification of Diseases, version 10, clinical modification.

# Supplementary Table 5. National Drug Codes (NDCs) used to identify statins.

| Medication | 9-Digit NDC |
| --- | --- |
| AMLODIPINE AND ATORVASTATIN | 000692150, 000692160, 000692170, 000692180, 000692190, 000692250, 000692260, 000692270, 000692960, 000692970, 000692980, 000696180, 000696323, 000696565, 000696747, 000697232, 000697476, 000697654, 000697810, 003784510, 003784511, 003784512, 003784513, 003784514, 003784515, 003784516, 003784517, 003784518, 003784519, 003784520, 003786161, 003786162, 003786163, 003786164, 003786165, 003786166, 003786167, 003786168, 003786169, 003786170, 003786171, 122800397, 122800398, 122800399, 435980313, 435980314, 435980315, 435980316, 435980317, 435980318, 435980319, 435980320, 435980321, 435980322, 435980323, 499990989, 545695704, 545695881, 545695951, 545696099, 548681207, 548683287, 548685179, 548685200, 548685209, 548685420, 548685523, 548685567, 548685672, 548685699, 548686335, 597626710, 597626711, 597626712, 597626720, 597626721, 597626722, 597626723, 597626730, 597626731, 597626732, 597626733, 605053478, 605053479, 605053483, 605053484, 605053488, 605053489, 605053492, 605053493, 633040499, |
| ASPIRIN AND PRAVASTATIN | 000035168, 000035169, 000035173, 000035174, 000035183, 000035184 |
| ATORVASTATIN | 000710155, 000710156, 000710157, 000710158, 000935056, 000935057, 000935058, 000935059, 001790141, 003782015, 003782017, 003782121, 003782122, 003783950, 003783951, 003783952, 003783953, 005913774, 005913775, 005913776, 005913777, 006158006, 006158007, 006158008, 006158009, 007815381, 007815382, 007815384, 007815388, 009046290, 009046291, 009046292, 009046293, 101350649, 101350650, 101350651, 101350653, 122800150, 134110113, 134110114, 134110115, 167140173, 167140174, 167140175, 167140176, 167140874, 167140875, 167140876, 167140877, 167290044, 167290045, 167290046, 167290047, 216950255, 332610959, 332610972, 332610973, 332610995, 333580210, 353560860, 353560894, 353560929, 422540019, 422540261, 422540267, 422540307, 422540379, 422540382, 422540391, 422540392, 422910143, 422910144, 422910145, 422910146, 425710172, 425710173, 425710174, 425710175, 427080003, 427080004, 427080005, 427080013, 427080014, 427080108, 427080141, 427080144, 430630373, 430630453, 430630474, 430630487, 430630496, |
| CERIVASTATIN | 000262883, 000262884, 000262885, 000262886, 545694589, 545694861, 545695180, 548684401, 548684436 |
| EZETIMIBE AND SIMVASTATIN | 001151385, 001151386, 001151387, 001151388, 122800181, 122800385, 122800386, 167140778, 167140779, 167140780, 167140781, 216950325, 216950339, 216950827, 435980583, 435980584, 435980585, 435980586, 435980742, 435980743, 435980744, 435980745, 459630565, 459630566, 459630567, 459630568, 499990957, 499990958, 500903984, 500904772, 511380363, 511380364, 511380365, 511380366, 514070190, 514070191, 514070192, 514070193, 545695648, 545695766, 545695768, 548685187, 548685189, 548685250, 548685259, 550480821, 550480822, 550480823, 552890280, 552890520, 552890980, 558870333, 558870882, 602191155, 602191156, 602191157, 602191158, 604290879, 604290880, 604290881, 604290882, 625590700, 625590701, 625590702, 625590703, 665820311, 665820312, 665820313, 665820315, 665820320, 665820321, 665820322, 665820323, 678770507, 678770508, 678770509, 678770510, 682586970, 682586984, 684620321, 684620322, 684620323, 684620324, 692381155, 692381156, 692381157, 692381158, 705181955, 782060174, 782060175, 782060176, |
| FLUVASTATIN | 000780176, 000780234, 000780354, 000937442, 000937443, 000937446, 003785121, 003788020, 003788021, 005272580, 007815370, 007818017, 134110111, 545693821, 545694761, 545695498, 548683329, 548684224, 548684601, 551753002, 552890476, 552890740, 636298737, 636298812, 661050147, 672630541, 675441029, 675441030 |
| LOVASTATIN | 000060730, 000060731, 000060732, 000930576, 000930926, 000930928, 001850070, 001850072, 001850074, 002282633, 002282634, 002282635, 002471129, 002471130, 003786510, 003786520, 003786540, 004406692, 004406693, 004406694, 004407692, 004407693, 004407694, 006158151, 006158152, 007811210, 007811213, 007811323, 009045581, 009045582, 009045583, 105440235, 105440241, 105440242, 105440246, 122800108, 165900547, 165900941, 216950534, 216950535, 216950536, 234905838, 234905839, 234905840, 332610547, 332610548, 332610549, 333580223, 333580224, 333580225, 333580226, 353560885, 422540025, 422540028, 422540106, 422910375, 422910376, 422910377, 425540028, 430630493, 430630548, 430630692, 430630731, 430630939, 430630983, 433530718, 433530763, 433530995, 459630633, 459630634, 459630635, 474634425, 474634442, 498840754, 498840755, 498840756, 499990293, 499990470, 499990471, 500900757, 500900759, 500900761, 500900762, 500902563, 500903216, 500903268, 500903396, 500903881, 502680510, 502680511, 502680512, |
| LOVASTATIN AND NIACIN | 000743005, 000743007, 000743010, 000743072, 548684807, 548684999, 548685087, 548685653, 605980006, 605980007, 605980008, 605980009 |
| PITAVASTATIN | 000024770, 000024771, 000024772, 252080200, 252080201, 252080202, 668690104, 668690204, 668690404, 707711117, 707711118 |
| PRAVASTATIN | 000030154, 000030178, 000030194, 000035154, 000035178, 000035194, 000035195, 000930771, 000937201, 000937202, 000937270, 002471139, 002471140, 002471276, 003780552, 003780553, 003780554, 003780557, 003788210, 003788220, 003788240, 003788280, 004408155, 004408156, 004408157, 004408158, 005910013, 005910014, 005910016, 005910019, 006158029, 006158030, 006158158, 006158159, 006158410, 007815231, 007815232, 007815234, 007815235, 009045891, 009045892, 009045893, 009046113, 009046114, 009046115, 101350498, 101350499, 101350500, 105440440, 105440505, 105440507, 122800038, 122800335, 134110118, 134110119, 162520526, 162520527, 162520528, 162520529, 165900546, 167140558, 167140559, 167140560, 167140570, 167290008, 167290009, 167290010, 167290011, 216950178, 216950179, 216950180, 234909350, 234909351, 234909352, 332610867, 332610868, 332610953, 353560125, 353560919, 353560921, 422540131, 422540202, 422540424, 422540425, 422540434, 422910665, 422910667, 422910668, 422910669, 425490490, 425490708, |
| ROSUVASTATIN | 000937570, 000937571, 000937572, 000937573, 003100751, 003100752, 003100754, 003100755, 003107560, 003107570, 003107580, 003107590, 003782201, 003782203, 003782204, 003782232, 007815400, 007815401, 007815402, 007815403, 009046602, 009046603, 009046604, 009046605, 009046778, 009046779, 009046780, 009046781, 122800164, 122800351, 136680179, 136680180, 136680181, 136680182, 162520615, 162520616, 162520617, 162520618, 165900411, 167140988, 167140989, 167140990, 167140991, 167290284, 167290285, 167290286, 167290287, 216950287, 216950288, 216950659, 216950759, 278080155, 278080156, 278080157, 278080158, 317220882, 317220883, 317220884, 317220885, 353560413, 353560519, 422910742, 422910743, 422910744, 422910745, 422920029, 422920030, 422920031, 422920032, 426770301, 426770302, 433530031, 433530245, 433530246, 433530249, 433530289, 433530290, 433530332, 433530339, 433530352, 435470591, 435470592, 435470593, 435470594, 473350582, 473350583, 473350584, 473350585, 473350984, 473350985, 473350986, |
| ROSUVASTATIN AND EZETIMIBE | 706610001, 706610002, 706610003, 706610004, 821200125, 821200126, 821200127, 821200128 |
| SIMVASTATIN | 000060543, 000060726, 000060735, 000060740, 000060749, 000937152, 000937153, 000937154, 000937155, 000937156, 001790128, 002471152, 002471153, 004062065, 004062066, 004062067, 004062068, 004062069, 004408320, 004408321, 004408322, 004408323, 004408324, 006157992, 006157993, 006158056, 007815070, 007815071, 007815072, 007815073, 007815074, 009045800, 009045801, 009045802, 101350508, 101350509, 101350510, 101350511, 101350512, 105440486, 105440487, 134110132, 134110133, 134110161, 134110162, 162520505, 162520506, 162520507, 162520508, 162520509, 165900431, 165900446, 165900726, 167140681, 167140682, 167140683, 167140684, 167140685, 167290004, 167290005, 167290006, 167290007, 167290156, 216950738, 216950739, 216950740, 216950741, 216950742, 234909353, 234909354, 234909355, 234909356, 234909357, 236290175, 236290176, 246580210, 246580211, 246580212, 246580213, 246580214, 246580300, 246580301, 246580302, 246580303, 246580304, 246580500, 246580501, 246580502, 246580503, 246580504, 292730401, |
| SIMVASTATIN AND NIACIN | 000743312, 000743315, 000743316, 000743455, 000743457, 000743459, 548685886, 548685904, 548685907, 548686169 |
| SIMVASTATIN AND SITAGLIPTIN | 000060533, 000060535, 000060537, 000060753, 000060757, 000060773 |
| AMLODIPINE AND ATORVASTATIN | 000692150, 000692160, 000692170, 000692180, 000692190, 000692250, 000692260, 000692270, 000692960, 000692970, 000692980, 000696180, 000696323, 000696565, 000696747, 000697232, 000697476, 000697654, 000697810, 003784510, 003784511, 003784512, 003784513, 003784514, 003784515, 003784516, 003784517, 003784518, 003784519, 003784520, 003786161, 003786162, 003786163, 003786164, 003786165, 003786166, 003786167, 003786168, 003786169, 003786170, 003786171, 122800397, 122800398, 122800399, 435980313, 435980314, 435980315, 435980316, 435980317, 435980318, 435980319, 435980320, 435980321, 435980322, 435980323, 499990989, 545695704, 545695881, 545695951, 545696099, 548681207, 548683287, 548685179, 548685200, 548685209, 548685420, 548685523, 548685567, 548685672, 548685699, 548686335, 597626710, 597626711, 597626712, 597626720, 597626721, 597626722, 597626723, 597626730, 597626731, 597626732, 597626733, 605053478, 605053479, 605053483, 605053484, 605053488, 605053489, 605053492, 605053493, 633040499, |

# Supplementary Table 6. International Classification of Diseases (ICD) codes used to identify patients with medical and psychiatric health conditions.

| Diagnosis | Coding System | Code |
| --- | --- | --- |
| Hypertension | ICD-9-CM | 401.0–401.9 |
|  | ICD-10-CM | I10 |
| Hypercholesterolemia | ICD-9-CM | 272.0 |
|  | ICD-10-CM | E78.00–E78.01 |
| Diabetes mellitus | ICD-9-CM | 250.00–250.91 |
|  | ICD-10-CM | E10.10–E11.9 |
| Chronic kidney disease, stage 3 or greater | ICD-9-CM | 585.3–585.6 |
|  | ICD-10-CM | N18.3–N18.6 |
| Depression | ICD-9-CM | 926.20–296.26,  296.30–296.36, 296.82, 296.99, 298.0, 300.4, 301.12, 311, 625.4 |
|  | ICD-10-CM | F32.0–F32.9, F33.0–F33.9,  F34.1 |
| Anxiety | ICD-9-CM | 300.00–300.09 |
|  | ICD-10-CM | F41.0–F41.9 |
| Bipolar disorder | ICD-9-CM | 296.00–296.06,  296.10–296.16,  296.40–296.46,  296.50–296.56,  296.60–296.66,  296.7, 296.80, 296.89 |
|  | ICD-10-CM | F30.1–F30.4, F31.0–F31.9 |
| Schizophrenia | ICD-9-CM | 295.00–295.05,  295.10–295.15,  295.20–295.25,  295.30–295.35,  295.40–295.45,  295.50–295.55,  295.60–295.65,  295.70–295.75,  295.80–295.85,  295.90–295.95 |
|  | ICD-10-CM | F20.0–F20.9, F25.9 |
| Alcohol use disorder | ICD-9-CM | 291.0–291.5,  291.5, 291.81–291.89, 291.9, 303.00–303.03,  303.90–303.93,  305.00–305.03 |
|  | ICD-10-CM | F10.10–F10.99 |
| Other substance use disorder | ICD-9-CM | 292.0, 292.11, 292.12, 292.2, 292,81–292.89, 292.9, 304.00–304.03,  304.10–304.13,  304.20–304.23,  304.30–304.33,  304.40–304.43,  304.50–304.53,  304.60–304.63,  304.70–304.72,  304.80–304.82,  304.90–304.92, 305.1, 305.20–305.23,  305.30–305.33,  305.40–305.43,  305.50–305.53,  305.60–305.63,  305.70–305.73,  305.80–305.83,  305.90–305.93 |
|  | ICD-10-CM | F11.10–F11.99,  F12.10–F12.99,  F13.10–F13.99,  F14.10–F14.99,  F15.10–F15.99,  F16.10–F16.99,  F17.200–F17.299,  F18.10–F18.99,  F19.10–F19.99 |

Abbreviations: ICD-9-CM; International Classification of Diseases, version 9, clinical modification; ICD-10-CM, International Classification of Diseases, version 10, clinical modification.

# Supplementary Table 7. International Classification of Diseases (ICD) codes and procedure codes used to identify patients with a history of cardiovascular disease.

| Diagnosis or Procedure | Coding System | Code |
| --- | --- | --- |
| Percutaneous revascularization | CPT | 92920–92944 |
| Coronary artery bypass graft | CPT | 33510–33536, 33572 |
| Acute myocardial infarction | ICD-9-CM | 410.0, 410.11, 410.21, 410.31, 410.41, 410.51, 410.61, 410.71, 410.81, 410.90–410.92 |
|  | ICD-10-CM | I21.01–I21.9 |
| Subsequent STEMI and NSTEMI | ICD-9-CM | 410.01, 410.11, 410.21, 410.31, 410.41, 410.51, 410.61, 410.71, 410.81, 410.91 |
|  | ICD-10-CM | I22.0–I22.9 |
| Certain current complications following STEMI and NSTEMI | ICD-9-CM | 429.5, 429.6, 429.71, 429.79 |
|  | ICD-10-CM | I23.0–I23.8 |
| Cerebral infarction | ICD-9-CM | 433.01, 433.11, 433.21, 433.31, 433.81, 433.91  434.01, 434.11, 434.91 |
|  | ICD-10-CM | I63.00–I63.9 |
| Occlusion and stenosis of precerebral arteries, not resulting in cerebral infarction | ICD-9-CM | 433.00, 433.10, 433.20, 433.80 433.90 |
|  | ICD-10-CM | I65.01–I65.9 |
| Cerebral atherosclerosis | ICD-9-CM | 437.0 |
|  | ICD-10-CM | I67.2 |
| Sequelae of cerebrovascular disease | ICD-9-CM | 438.0–438.9 |
|  | ICD-10-CM | I69.00–I69.998 |
| Presence of aortocoronary bypass graft | ICD-9-CM | V45.81 |
|  | ICD-10-CM | Z95.1 |
| Presence of coronary angioplasty implant and graft | ICD-9-CM | V45.82 |
|  | ICD-10-CM | Z95.5 |

Abbreviations: CPT, Current Procedural Terminology; HCPCS, Healthcare Common Procedure Coding System; ICD-9-CM; International Classification of Diseases, version 9, clinical modification; ICD-10-CM, International Classification of Diseases, version 10, clinical modification.

# Supplementary Table 8. Characteristics associated with statin discontinuation and low statin adherence among 9522 people with HIV initiating a statin, MarketScan Commercial Claims and Encounters Databases, 2015–2022.

|  | Discontinuation* | Low Adherence^†^ |
| --- | --- | --- |
| Characteristic | Unadjusted HR (95% CI) ^‡^ | Unadjusted PR (95% CI) ^§^ |
| Women vs. men | 1.33 (1.23, 1.44) | 1.36 (1.29, 1.44) |
| Age, years |  |  |
| Quartile 1 (<46) | 1.37 (1.24, 1.50) | 1.43 (1.34, 1.52) |
| Quartile 2 (46–52) | 1.14 (1.04, 1.25) | 1.14 (1.07, 1.22) |
| Quartile 3 (53–57) | 1.05 (0.95, 1.15) | 1.05 (0.99, 1.12) |
| Quartile 4 (≥58) | 1 (ref.) | 1 (ref.) |
| Region of residence |  |  |
| Northeast | 0.84 (0.76, 0.92) | 0.96 (0.89, 1.02) |
| North Central | 0.76 (0.68, 0.85) | 0.80 (0.73, 0.87) |
| South | 1 (ref.) | 1 (ref.) |
| West | 0.70 (0.63, 0.77) | 0.75 (0.69, 0.80) |
| Calendar year |  |  |
| 2015–2017 | 1.14 (1.05, 1.24) | 0.94 (0.89, 1.00) |
| 2018–2019 | 1.01 (0.93, 1.10) | 0.90 (0.85, 0.95) |
| 2020–2021 | 1 (ref.) | 1 (ref.) |
| Hypertension | 0.99 (0.93, 1.05) | 0.97 (0.93, 1.02) |
| Hypercholesterolemia | 0.89 (0.80, 0.99) | 0.85 (0.79, 0.90) |
| Diabetes | 1.01 (0.94, 1.10) | 1.03 (0.98, 1.09) |
| CKD, stage ≥3 | 0.88 (0.75, 1.04) | 0.94 (0.85, 1.04) |
| Mental health disorder | 0.85 (0.79, 0.92) | 0.92 (0.87, 0.96) |
| Alcohol use disorder | 1.01 (0.83, 1.24) | 1.07 (0.95, 1.20) |
| Other substance use disorder | 1.02 (0.92, 1.12) | 1.04 (0.98, 1.11) |
| PI/COBI use | 0.97 (0.91, 1.04) | 0.96 (0.92, 1.01) |
| Statin intensity |  |  |
| Low | 1 (ref.) | 1 (ref.) |
| Medium | 0.93 (0.85, 1.01) | 0.98 (0.92, 1.04) |
| High | 1.03 (0.93, 1.15) | 1.08 (1.00, 1.17) |

Abbreviations: CI, confidence interval; CKD, chronic kidney disease; COBI, cobicistat; HR, hazard ratio; PI, protease inhibitor; PR, prevalence ratio; ref., referent.

* First gap in statin use >90 days.

^†^ Low adherence was defined as having a 90-day proportion days covered (PDC) <80%.

^‡^ Estimated from separate Cox proportional hazards models for each variable, measured at statin initiation.

^§^ Estimated from separate log-binomial regression models for each variable, with generalized estimating equations accounting for patients contributing more than one 90-day interval to the analysis. Each variable is measured at the start of every 90-day interval.

# Supplementary Table 9. Sensitivity analyses using different thresholds of proportion of days covered (PDC) to define low adherence.

|  | PDC <90% | | | PDC <70% | | |
| --- | --- | --- | --- | --- | --- | --- |
| Gender | Probability of low adherence ^§^ | Unadjusted PR (95% CI) * | Adjusted PR (95% CI) *^, †^ | Probability of low adherence ^§^ | Unadjusted PR (95% CI) * | Adjusted PR (95% CI) *^, †^ |
| Women | 40.5% (38.7%, 42.4%) | 1.33 (1.26, 1.40) | 1.27 (1.21, 1.33) | 29.3% (27.7%, 30.9%) | 1.37 (1.28, 1.45) | 1.29 (1.22, 1.37) |
| Men | 30.5% (29.8%, 31.3%) | 1 (ref.) | 1 (ref.) | 21.4% (20.8%, 22.1%) | 1 (ref.) | 1 (ref.) |

Abbreviations: CI, confidence interval; PR, prevalence ratio; ref., referent.

* Estimated from a log-binomial regression model with generalized estimating equations accounting for patients contributing more than one 90-day interval to the analysis.

^†^ Adjusted for calendar year, US census region, employment status, and statin intensity, measured at statin initiation, and for time-updated age, use of a protease inhibitor or cobicistat-boosted antiretroviral therapy regimen, and history of hypertension, hypercholesterolemia, diabetes mellitus, chronic kidney disease stage 3 or greater, mental health disorders, alcohol use disorder, and other substance use disorder, measured at the start of every 90-day interval.

# Supplementary Table 10. Sensitivity analysis censoring all people with HIV after three years since statin initiation.

| Gender | Two-year risk of discontinuation * | Unadjusted HR (95% CI) ^†^ | Adjusted HR (95% CI) ^†, ‡^ |
| --- | --- | --- | --- |
| Women | 59.0% (55.9%, 62.2%) | 1.34 (1.23, 1.45) | 1.31 (1.20, 1.42) |
| Men | 48.1% (46.7%, 49.5%) | 1 (ref.) | 1 (ref.) |
| Gender | Probability of low adherence ^§^ | Unadjusted PR (95% CI) ^\|\|^ | Adjusted PR (95% CI) ^\|\|, ¶^ |
| Women | 35.3% (33.7%, 37.0%) | 1.34 (1.27, 1.41) | 1.28 (1.22, 1.35) |
| Men | 26.4% (25.8%, 27.1%) | 1 (ref.) | 1 (ref.) |

Abbreviations: CI, confidence interval; HR, hazard ratio; PR, prevalence ratio; ref., referent.

* First gap in statin use >90 days.

^†^ Estimated from a Cox proportional hazards model.

^‡^ Adjusted for age, calendar year, US census region, statin intensity, use of a protease inhibitor or cobicistat-boosted antiretroviral therapy regimen, and history of hypertension, hypercholesterolemia, diabetes mellitus, chronic kidney disease stage 3 or greater, mental health disorders, alcohol use disorder, and other substance use disorder, all measured at statin initiation.

^§^ Low adherence was defined as having a 90-day proportion days covered (PDC) <80%.

^||^ Estimated from a log-binomial regression model with generalized estimating equations accounting for patients contributing more than one 90-day interval to the analysis.

^¶^ Adjusted for calendar year, US census region, employment status, and statin intensity, measured at statin initiation, and for time-updated age, use of a protease inhibitor or cobicistat-boosted antiretroviral therapy regimen, and history of hypertension, hypercholesterolemia, diabetes mellitus, chronic kidney disease stage 3 or greater, mental health disorders, alcohol use disorder, and other substance use disorder, measured at the start of every 90-day interval.

# References

1. Alonso A, Barnes AE, Guest JL, et al. HIV Infection and Incidence of Cardiovascular Diseases: An Analysis of a Large Healthcare Database. *J Am Heart Assoc*. 2019;8(14):e012241.

2. Crockett KB, Wen Y, Overton ET, et al. One-year statin persistence and adherence in adults with HIV in the United States. *J Clin Lipidol*. 2021;15(1):181–191.

3. Gallant J, Hsue PY, Shreay S, et al. Comorbidities Among US Patients With Prevalent HIV Infection-A Trend Analysis. *J Infect Dis*. 2017;216(12):1525–1533.

4. King H, Bull-Otterson L, Hoover KW, et al. Factors Associated With Testing for Hepatitis C Infections Among a Commercially Insured Population of Persons With HIV, United States 2008-2016. *Open Forum Infect Dis*. 2020;7(6):ofaa222.

5. Kong AM, Pozen A, Anastos K, et al. Non-HIV Comorbid Conditions and Polypharmacy Among People Living with HIV Age 65 or Older Compared with HIV-Negative Individuals Age 65 or Older in the United States: A Retrospective Claims-Based Analysis. *AIDS Patient Care STDS*. 2019;33(3):93–103.

6. Rosenson RS, Hubbard D, Monda KL, et al. Excess Risk for Atherosclerotic Cardiovascular Outcomes Among US Adults With HIV in the Current Era. *J Am Heart Assoc*. 2020;9(1):e013744.

7. Siddiqui J, Samuel SK, Hayward B, et al. HIV-associated wasting prevalence in the era of modern antiretroviral therapy. *AIDS*. 2022;36(1):127–135.

8. Siddiqui M, Hannon L, Wang Z, et al. Hypertension and Cardiovascular Disease Risk Among Individuals With Versus Without HIV. *Hypertension*. 2023;80(4):852–860.
